# Calcium Overload and Mitochondrial Metabolism

**DOI:** 10.3390/biom12121891

**Published:** 2022-12-17

**Authors:** Lauren L. Walkon, Jasiel O. Strubbe-Rivera, Jason N. Bazil

**Affiliations:** 1 Department of Physiology, Michigan State University, East Lansing, MI 48824, USA; 2 Department of Pharmacology and Toxicology, Michigan State University, East Lansing, MI 48824, USA

**Keywords:** bioenergetics, calcium overload, mitochondria, mitochondrial ultrastructure, mitochondrial function, oxidative phosphorylation, mitochondrial ATP production, calcium phosphate, calcium precipitates

## Abstract

Mitochondria calcium is a double-edged sword. While low levels of calcium are essential to maintain optimal rates of ATP production, extreme levels of calcium overcoming the mitochondrial calcium retention capacity leads to loss of mitochondrial function. In moderate amounts, however, ATP synthesis rates are inhibited in a calcium-titratable manner. While the consequences of extreme calcium overload are well-known, the effects on mitochondrial function in the moderately loaded range remain enigmatic. These observations are associated with changes in the mitochondria ultrastructure and cristae network. The present mini review/perspective follows up on previous studies using well-established cryo–electron microscopy and poses an explanation for the observable depressed ATP synthesis rates in mitochondria during calcium-overloaded states. The results presented herein suggest that the inhibition of oxidative phosphorylation is not caused by a direct decoupling of energy metabolism via the opening of a calcium-sensitive, proteinaceous pore but rather a separate but related calcium-dependent phenomenon. Such inhibition during calcium-overloaded states points towards mitochondrial ultrastructural modifications, enzyme activity changes, or an interplay between both events.

## 1. Introduction: Mitochondrial Calcium—The Good and the Bad

**Figure 1:**
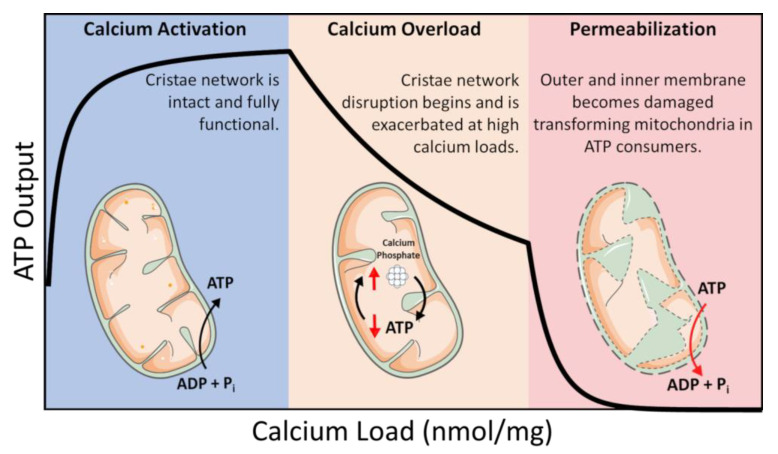
Calcium overload. In low amounts, calcium enhances mitochondrial function by activating several Ca^2+^–sensitive catabolic enzymes. In moderate amounts, depressed rates of oxidative phosphorylation become observable. In extreme amounts, mitochondria become structurally compromised and consume ATP in a futile attempt to restore homeostasis.

ATP is coupled to nearly every reaction in the body and is necessary for an organism’s survival. This essential energy metabolite is primarily produced by mitochondria in a process known as oxidative phosphorylation. Oxidative phosphorylation is regulated in a manner that ensures the optimal rate of mitochondrial ATP production. ATP breakdown products, ADP, and inorganic phosphate (Pi) are the most potent regulators of oxidative phosphorylation. That said, calcium is also an important regulator but acts as a double–edged sword regarding oxidative phosphorylation [1,2]. Calcium ions enter and leave mitochondria through a variety of specialized channels and transporters in a tissue-specific manner [3]. Moreover, mitochondria possess a unique ability to accumulate massive quantities of calcium in their matrix with devastating consequences [1,2,4,5]. While relatively low amounts of calcium (0 < 40 nmol/mg mitochondria) are essential for energy production, high levels of calcium (>500 nmol/mg) lead to the total collapse of energy homeostasis (Figure 1) [6]. In between, a state known as calcium overload (40–500 nmol/mg), calcium impairs oxidative phosphorylation and presumably contributes to long–term organ dysfunction. These ranges were identified using guinea pig cardiac mitochondria, and external effectors such as cyclosporin A (CsA) can modulate them [2,7]. Thus, the regulation of mitochondrial calcium content is of utmost importance for living tissue.

In this intermediate calcium overloaded state, ATP synthesis is inhibited by total mitochondrial calcium in a titratable manner [1,2,8]. This inhibition is relieved after calcium is removed from mitochondria if the total content is below 500 nmol/mg, and calcium removal only partially recovers ATP synthesis capacity at higher loads [5]. Phosphate facilitates mitochondrial calcium uptake but can ultimately lead to cell death via the mitochondrial permeability transition phenomenon [9,10]. While phosphate is thought to act as a permeability transition inducer, it may also serve a dual purpose as a desensitizer under certain conditions [11]. While the molecular details of the mitochondrial permeability transition phenomenon are still debated, the current consensus revolves around the idea of the formation of a proteinaceous pore [12,13]. That said, the conditions required to open this pore in vitro are extreme and would result in irreversible cell death in vivo. Thus, it may not play a significant role in how calcium regulates energy metabolism in living tissue. There are numerous review articles discussing our current understanding of this phenomenon [14,15]. Discussed herein, and previously by our group [2,5], is an emerging idea that views this phenomenon from a different perspective which involves a conceptual link between mitochondrial ultrastructure and function.

## 2. Materials and Methods

Mitochondria isolation and protein quantification. Cardiac mitochondria were obtained from Hartley albino guinea pig hearts weighting 350–450 g (4–6 weeks old). The animals were injected with heparin (500 units/mL) in the intraperitoneal cavity and subjected to anesthesia with 4–5% isoflurane prior to guillotine decapitation. The heart was obtained following a thoracotomy procedure and perfused with a cold cardioplegia solution. The heart tissue was minced into ~10 mm pieces and homogenized using a handheld homogenizer at 18,000 rpm for 20 s. Mitochondria were isolated using differential centrifugation as described in [2,5,7]. The mitochondrial protein quantification was performed using the BIO-RAD bovine serum albumin (BSA) standard set kit and the bicinchoninic acid (BCA) assay. The mitochondrial suspension was diluted to 40 mg/mL and kept on ice throughout the duration of the experiments (4–5 h).

Mitochondrial quality control. The mitochondrial respiratory control ratio (RCR) was determined using an Oxygraph 2k (Oroboros Instrument Corp., Innsbruck, Austria) by loading 2 mL of a respiratory buffer containing 130 mM KCl, 5 mM K_2_HPO_4_, 20 mM 3-(N-morpholino) propanesulfonic acid (MOPS), 1 mM MgCl_2_, 1 mM ethylene glycol-bis(β-aminoethyl ether)-N,N,N′,N′-tetraacetic acid (EGTA), and 0.1% (*w*/*v*) BSA at a pH of 7.1 and 37 °C. Before the addition of 0.1 mg/mL mitochondria, 5 mM sodium pyruvate and 1 mM L–malate (pH 7.0) were added. All the following experiments were performed using the described conditions and buffer. Following the addition of 0.1 mg/mL mitochondria. the leak state was recorded for 5 min. Here, we define leak state as the rate of oxygen consumption by the mitochondria in the presence of substrate and absence of ADP. At 5 min, a bolus of 500 µM ADP was added to determine the maximum ADP-stimulated respiratory rate. The mitochondrial quality was assessed by computing the RCR obtained by dividing the maximal ADP–stimulated respiration rate by the leak respiration rate. Only Mitochondria with RCR values greater than or equal to 16 met the quality criteria and used for experiments.

Calcium effects on mitochondrial respiration. To assess the effects of calcium on mitochondrial function, leak state was recorded after the addition of 5 mM sodium pyruvate and 1 mM L–malate and 0.1 mg/mL mitochondria. At 5 min, water vehicle, 25 µM CaCl_2_, or a 50 µM CaCl_2_ bolus was injected into the oxygraphy chamber. For the zero Ca^2+^ conditions, 1 mM EGTA was present throughout the experiment. When the water vehicle is used, it is important to note that 4 µM residual Ca^2+^ from buffer contaminants is initially present. At 10 min, a 500 µM ADP bolus was injected to induced maximal ADP-stimulated respiration rates.

Cryo–electron microscopy (cryo–EM) sample vitrification and tomographic acquisition. Isolated mitochondria were suspended at a concentration of 0.1 mg/mL in 2 mL of respiration buffer containing 5 mM sodium pyruvate and 1 mM L–malate. At 5 min, water vehicle, 1 mM EGTA, 25 µM CaCl_2_, or a 50 µM CaCl_2_ bolus was injected into the oxygraphy chamber. For the cyclosporin A (CsA) treatment, 1µM CsA was added to the suspension before the addition of mitochondria. At 10 min, 5 µL samples were pipetted from the mitochondrial suspension and deposited on Quantifoil R2/2 holey carbon grids pretreated with a Pelco EasiGlo glow discharge unit for 1 min. The grids were set on a Vitrobot Mark IV chamber with automated temperature regulation (4 °C), blotting (3 s), and humidity control (100%). Samples were blotted to thin the water layer, plunged into liquid ethane, then transferred and stored in liquid nitrogen until imaging. The imaging and tomographic acquisition was collected using a FEI Talos Artica at 200 keV in low-dose conditions on a Falcon 3EC direct electron detector with an electron dose of ~2 e^−^/Å^2^ per tilt image. The tomographic images were collected at 22,000 x magnification obtaining a final product of 4.7 Å/pixel with a total electron dose of ~100 e^−^/Å^2^.

Tomogram alignment and 3D reconstruction. Motion correction was performed on each individual micrograph using Motioncor2 v1.2.6 with an index factor of 7. The tilt series alignment was performed using IMOD v4.9.12 and the Simultaneous Iterative Reconstruction Technique (SIRT) feature with 7–10 iterations. The 3D reconstruction tracings were performed using IMOD (3dmod) drawing tool functionality [16].

Statistics. All data were analyzed and plotted using MATLAB 2022a (Mathworks, Inc., Natick, MA, USA). The data are presented as a mean standard deviation for a sample size of *n* ≥ 8.

## 3. Calcium Homeostasis, Entry, and Exit Pathways

Mitochondrial calcium homeostasis is primarily regulated by three pathways: the mitochondrial calcium uniporter (MCU), sodium/calcium/lithium exchanger (NCLX), and calcium hydrogen exchanger (CHE). The MCU is the dominant uptake pathway and is comprised of a heteromeric protein complex composed of various subunits including the mitochondrial Ca^2+^ uptake family (MICU 1, 2, and 3), essential MCU regulator (EMRE), MCU regulator 1 (MCUR1), MCU dominant-negative β–subunit (MCUb), and the solute carrier 25A23 (SLC25A23) [17]. These subunits form a complex on what appears to be an on-demand basis [17,18,19,20]. While an in–depth review on the molecular structure and function of these subunits are beyond the scope of this mini review/perspective, we would like to refer our readers to references [17,19,20]. Other pathways for Ca^2+^ uptake including the rapid mode calcium uptake and ryanodine receptors are also speculated to play an important role under certain conditions [21,22,23]. The MCU channel has a high affinity for Ca^2+^ (Kd ≤ 2 nM); however, it has a low half-activation constant (K_0.5_~20 mM) [24]. As a result, the MCU is less active at lower concentrations of calcium (0.1–1 µM) and more active at higher extramitochondrial calcium loads [21]. The efflux pathways are primarily controlled by the NCLX and CHE. The NCLX is an electrogenic exchanger that swaps 3 Na^+^ (or Li^+^) for 1 Ca^2+^ [25,26]. Consequently, this reaction is electrogenic and sensitive to the mitochondrial membrane potential. Under physiological conditions, the CHE swaps calcium out for protons in the matrix in a manner that is presumed to be an electroneutral ratio of 1 Ca^2+^ per 2 H^+^ [27]. The calcium hydrogen exchanger functions independently of sodium and is present at much lower activities in tissues with high energy demand [28]. For instance, the CHE is dominant in the liver and other relatively quiescent tissues, while NCLX is predominant in the heart, brain, and other high activity tissues [29,30,31,32,33,34].

Under basal conditions, cytosolic Ca^2+^ concentrations are maintained in the 100 nM range [35]. For cardiomyocytes and skeletal myocytes, the range of global or average cytosolic Ca^2+^ concentrations during peak contraction that mitochondria are exposed to fall within 1 µM but can peak two to three times higher under stimulatory conditions [36]. That said, some mitochondria are exposed to higher concentrations (~10–100 µM) in microdomains associated with mitochondrial–SR contact sites [37,38,39]. In either the basal or stimulatory condition, intramitochondrial Ca^2+^ levels remain low as long as mitochondria remain coupled [37]. This form of calcium regulation is attributed to the mitochondrial calcium buffering system. What makes mitochondria particularly relevant in this scenario is their ability to store large amounts of calcium in their matrix [40]. 

This is of relevance in high–energy demand tissue as the mitochondrial membrane potential, Ca^2+^, and Na^+^ are the main regulators of mitochondrial calcium homeostasis. Additionally, while Ca^2+^ uptake is very sensitive to changes in membrane potential, Ca^2+^ efflux is less sensitive [41]. As a result, the MCU channel can load Ca^2+^ into the matrix at a rate far exceeding the NCLX matrix calcium clearance (1400 and 20 nmol Ca^2+^ min^−1^ mg mitochondrial protein^−1^, respectively) [42]. Hence, under conditions that disrupt cytosolic calcium homeostasis, Ca^2+^ uptake through MCU floods the matrix of energized mitochondria with massive amounts of Ca^2+^ [8]. Left unchecked, membrane potential loss precipitates a catastrophic collapse in energy homeostasis [2,43]. This phenomenon is often ascribed to the permeability transition phenomenon and has detrimental consequences for cell health and longevity [4,9,15,42,44]. This scenario places mitochondria in a vulnerable position, leading some to view the permeability transition phenomenon as a calcium overload release valve [45,46]. Regardless, the mitochondria Ca^2+^ uptake and removal processes are highly regulated with compensatory mechanisms in place to ensure cellular homeostasis and survivability. When such regulatory mechanisms fail, mitochondria become overloaded with calcium, and energy homeostasis collapses.

## 4. Calcium TCA and ETC

Calcium also influences mechanisms driving mitochondrial energy production and metabolic activity. For example, calcium regulates the activity of metabolic enzymes in the TCA cycle including pyruvate dehydrogenase, isocitrate dehydrogenase, and α–ketoglutarate dehydrogenase [2]. The TCA cycle generates reducing equivalents (such as NADH and UQH_2_) used by the proton pumps that establish the membrane potential and alkalize the mitochondrial matrix relative to the cytoplasm [26]. This, in turn, biases ATP synthase away from its more favorable ATP hydrolysis set point to an operating regime conducive for ATP production via oxidative phosphorylation. Therefore, in high–energy demanding tissue where cells are constantly exposed to transient Ca^2+^ signals, calcium homeostasis is intrinsically linked to ATP production both through the TCA cycle and oxidative phosphorylation (oxphos) to regulate cellular bioenergetics.

Under most conditions, the membrane potential is the primary determinant of the ratio of matrix ATP to ADP and inorganic phosphate (i.e., the matrix ATP/ADP/Pi ratio). As Ca^2+^ is injected into mitochondria, matrix ATP/ADP ratios decline, the membrane potential depolarizes to a degree, and matrix pH increases [44,47]. As the matrix pH becomes more basic, dihydrogen phosphate (H_2_PO_4_^−^) is effectively driven into the mitochondria in symport with H^+^ in an electroneutral fashion. The phosphate carrier facilitates his symport, and when H_2_PO_4_^−^ enters the matrix, it undergoes a deprotonation event and forms HPO_4_^2−^ [48]. Under these conditions, elevated Pi levels in the matrix facilitate the formation of calcium phosphate complexes. The formation of these complexes involves the deprotonation of HPO_4_^2−^ into the phosphate trianion (PO_4_^3−^) further releasing a H^+^. This helps counteract the significant alkalizing effect of accelerated proton pumping and charge replacement caused by Ca^2+^ uptake. Overall, a slight depolarization will alkalize the matrix pH which has the net effect of enhancing mitochondrial Ca^2+^ sequestration [43]. However, when the current generated by Ca^2+^ uptake exceeds the proton pumping current, thermodynamic driving forces reverse the F_1_F_O_ ATP synthase activity and pumps protons out of the matrix via ATP hydrolysis [6]. When ATP is hydrolyzed from this reversal, the phosphate released can participate in phosphate precipitate formation until ATP is exhausted and the metabolic system collapses [40,49]. This is just one possible scenario as ATP hydrolysis is not a required source of inorganic phosphate during precipitate formation. In the presence of oligomycin, ATP synthase is inhibited, and mitochondria still possess the ability to take up massive amounts of Ca^2+^ when sufficient Pi is present [50]. Ultimately, when matrix Ca^2+^ concentrations exceed a threshold level, the formation of calcium phosphate precipitates in the matrix has the effect of reducing the mitochondrial free Ca^2+^ levels to manageable amounts via a type of buffering mechanism but at the expense of oxidative phosphorylation capacity [1,21].

## 5. Mitochondrial Calcium Buffering

We know that the consequences of precipitate formation operate on a spectrum (Figure 1), but we do not fully comprehend the mechanism. At low concentrations, calcium phosphate precipitates can have a protective effect. Whereas at high concentrations, precipitates can destabilize the mitochondrial cristae network [2,5]. This has been confirmed by others in which mitochondria loaded with Ca^2+^ resulted in calcium phosphate precipitates occupying more than 20% of the matrix volume [2]. One potential mechanism that leads to metabolic dysfunction is that precipitates may mechanically destabilize membrane structures by disrupting proteins involved in maintaining the cristae structure [5]. Another idea is that precipitates may serve as physical barriers limiting metabolite and substrate diffusion across the matrix [1]. The regulatory characteristics of the phosphate precipitation buffering mechanism remain enigmatic, but one concept boils down precipitate formation to a simple thermodynamic argument [40,49]. A second concept includes the idea that precipitate formation requires nucleation sites [51]. Full occupancy of these sites might dampen the extent of phosphate buffering within the mitochondrial matrix and send free Ca^2+^ high into pathological concentrations. The unknown nature of these potential nucleation sites makes it challenging to devise effective genetic and pharmacological approaches to manipulate mitochondrial calcium buffering. Thus, further study is required to resolve some of these unknowns.

## 6. Potential Role of Annexins in Mitochondria

Under appropriate conditions, when the mitochondria are energized, and magnesium and phosphate are present, adenine nucleotides are taken up with Ca^2+^ during precipitate formation [52]. However, the mechanism as to how precipitate formation is accomplished is yet to be elucidated but may be linked to annexins [53,54]. These proteins consist of a multigene family of Ca^2+^–regulated proteins with a calcium and lipid–binding modules known as the core domain. Some even possess GTP/ATP binding capabilities that enhance Ca^2+^/lipid interactions [55,56]. Their ubiquitous nature covers a variety of cellular functions including membrane transport, membrane–domain organization, anti-inflammatory and fibrinolytic activities, membrane repair, Ca^2+^ signaling pathways and even mitochondrial morphogenesis [57,58,59,60]. Others have theorized that annexins can act as a “lipid patch” to aid in injury repair [61]. Additionally, while the various annexin affinities for Ca^2+^ are different, it was demonstrated that increased annexin Ca^2+^ binding is correlated with plasma membrane repair [62]. Initially, annexins most sensitive to Ca^2+^ are bound and as the healing process proceeds, Ca^2+^ concentrations increase and the annexins presented are less sensitive to Ca^2+^. That said, annexins may also bind to the inner membrane and form nucleation sites for precipitate growth [53]. In doing so, annexins can reduce free Ca^2+^ concentrations and prevent the activation of Ca^2+^–dependent degradation processes in the matrix. Hence, annexins may be involved in signaling related to mitochondrial calcium overload, but the extent of which is currently unknown. Perhaps the biggest question is whether annexins are linked to the permeability transition phenomenon or the Ca^2+^ buffering system. Hence, functional studies, coupled with structural assessments, looking at the expression and activity of annexins with respect to Ca^2+^ handling in cardiac mitochondria could prove fruitful. However, the connection between mitochondrial ultrastructure and energy transduction is an emerging field. That said, we are still quite limited today, but cryo–EM has shed new light on the subject.

## 7. Structure/Function Axis

Mitochondrial ultrastructure undergoes dramatic changes during metabolic perturbations or in the presence of certain genetic modifications [63,64,65,66,67,68,69,70]. As such, calcium overload is a way to induce mitochondrial structural modifications. The response of mitochondria to Ca^2+^ was first reported about a half-century ago and was shown to decouple mitochondrial ATP production in extremely overloaded states via the permeability transition phenomenon [71,72,73,74,75,76,77,78,79,80,81,82]. A different perspective on the matter involves the incorporation of structural information with coincident function data which is summarized in Figure 1. The idea linking structure to function is not new; however, the effect of Ca^2+^ on ultrastructure is novel and warrants further investigation. The importance of this concept is borne out through two simple facts. The first involves matrix contracture following ADP binding [83]. This presumably enhances energy transduction, a theory yet to be experimentally or computationally verified. The second encompasses ultrastructural changes induced by the presence of excess Ca^2+^ [2,84,85]. From this mechanism, the intriguing phenotypes reported in prior work can be explained by a metabolic flux imbalance caused by Ca^2+^-induced cristae network disruption via metabolite permeability changes induced by cristae junction modifications.

**Figure 2:**
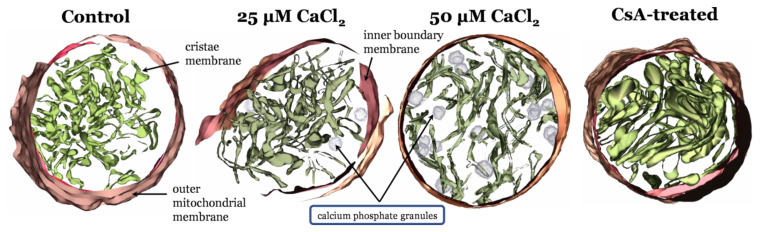
Mitochondrial ultrastructural changes associated with calcium overload. From left to right, IMOD [16] 3D mitochondrial reconstructions from cryo–electron tomography data where mitochondria were exposed to zero Ca^2+^, a bolus of 25 µM CaCl_2_, a bolus of 50 µM CaCl_2_ [2], and 1µM CsA. Calcium causes a decrease in cristae volume in a titratable manner. CsA leads to an expanded cristae volume and altered outer membrane morphology.

Following up on our previous modeling study [1], we have identified novel structural changes associated with calcium overload and treatments known to protect against its devastating effects [5]. We hypothesize that differential cristae junctional protein processing underly the differences in these phenotypes. Figure 2 shows that as the Ca^2+^ load increases, the ultrastructural changes become more and more pronounced. These changes in ultrastructure are responsible for depressed ATP synthesis rates [7]. In the calcium overloaded state, the cristae network becomes “stringy”, and the matrix volume expands with embedded Ca^2+^ phosphate precipitates located within the matrix near cristae junctions [2]. Intriguingly, the impact of cyclosporin A (CsA) on ultrastructure aligns with prior work which shows that this compound causes cristae membrane condensation and enhances metabolic flux [5,86]. These morphological changes are independent of the permeability transition pore [7] and require new approaches capable of demystifying the links between membrane morphology and energy transduction.

While these images reveal a striking effect of calcium and CsA on the ultrastructure of isolated mitochondria, they only provide possible explanations for the observed reduction of maximum rates in oxidative phosphorylation in the calcium overloaded state. One clue lies within the O2 respiratory dynamics during oxidative phosphorylation in the various conditions.

## 8. Oxygen Utilization in the Calcium Overloaded State

**Figure 3:**
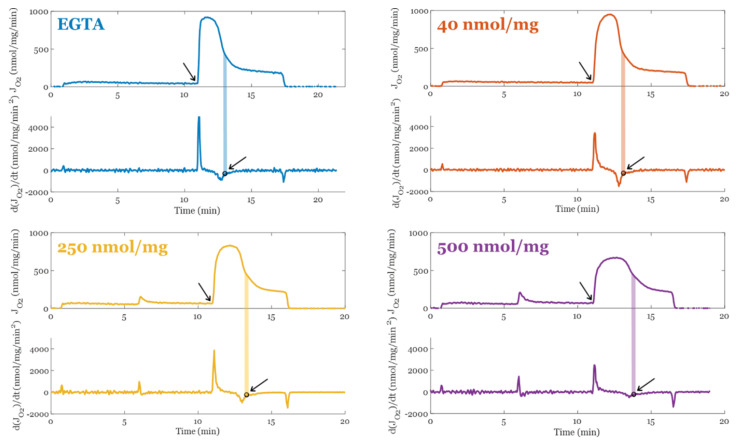
Method used to calculate the duration of oxidative phosphorylation. The transition is marked with an open circle and a shaded area for each condition that was used to estimate the point the majority of the O_2_ flux switches from oxphos using the original ADP bolus to futile ATP cycling. This ATP cycling occurs when the rate of mitochondrial ATP efflux matches the ATP hydrolysis rate from extramitochondrial ATPase contaminants. All mitochondrial preparations when Mg^2+^ is present contain these contaminants. The arrows point to the start and end of oxphos for each condition.

Prior studies have identified that when mitochondria are in a calcium overloaded state, their ability to oxidatively phosphorylate ADP is compromised [7,87]. This could be due to transient permeability transition events which decouples proton pumping from ATP synthesis. Alternatively, ultrastructural changes could underly the altered respiratory dynamics that occur during oxidative phosphorylation. One way to test this is to estimate the amount of O_2_ utilized per ADP phosphorylated. Estimating these values is difficult and requires the right protocols. Since we collected our data with a different objective in mind, we developed a suitable alternative approach. Using the information of the time derivative of the respiratory rate (J_O2_), we estimated the duration of oxidative phosphorylation in a manner that is robust against experimental condition. The method is summarized in Figure 3. At the time point when oxidative phosphorylation is winding down, there is a transition that marks when the system is entering its final approach to a new steady state. This method was robust with respect to environmental conditions and produced ATP/O_2_ ratios close to the theoretical value. That said, the exact time or transition point in the vicinity of our selection is not critical if the respiratory phase or transition is the same between conditions.

**Figure 4:**
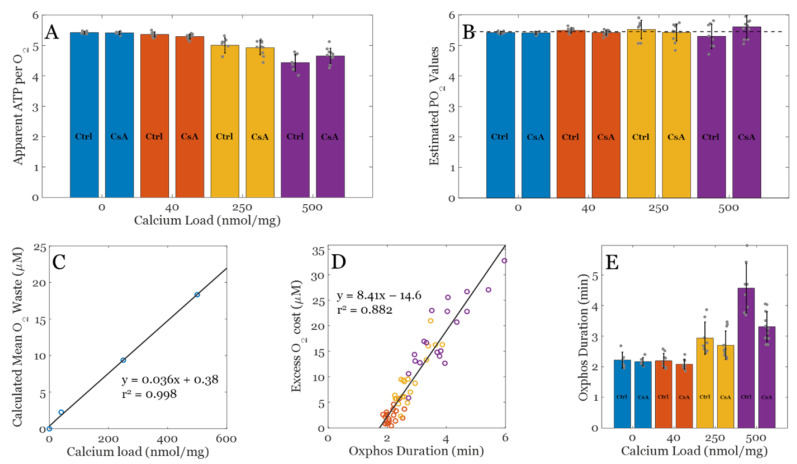
Analysis of O_2_ cost for each experimental condition. (**A**) Observed ADP consumed (ATP produced) per O_2_ across a range of calcium loads with and without CsA. (**B**) Estimated ATP/O_2_ ratios across a range of calcium loads with and without CsA. The theoretical PO_2_ value for NADH linked substrates is approximately 5.46 assuming 8/3 + 1 H^+^ per ATP generated and exported and 20 H^+^ per O_2_ consumed. (**C**) Calculated mean O_2_ waste during the oxphos period used to correct apparent ADP per O_2_ data shown in panel A to estimate the PO_2_ values shown in panel B. (**D**) Calculated excess O_2_ cost plotted against oxphos duration from panel C. (**E**) Oxphos duration estimated from data for each condition. Color key: blue, EGTA; orange, 40 nmol/mg, yellow, 250 nmol/mg, and 500 nmol/mg Ca^2+^ condition. Data are presented as the mean ± standard deviation for a sample size of *n* ≥ 8.

Figure 4 summarizes the effect of calcium overload on oxygen utilization during oxidative phosphorylation in the absence and presence of CsA. The apparent ATP produced per O_2_ consumed for each condition are shown in Figure 4A. This was calculated based on a total ADP bolus of 500 µM and assuming enough is converted to ATP before ATP cycling occurs at an appreciable rate so that a reliable estimate of O_2_ cost may be calculated. CsA has little to no effect on these values; therefore, the permeability transition phenomenon is not relevant here. As the Ca^2+^ load increases, fewer ATP molecules are produced from the same number of O_2_ molecules. This occurs because background cation cycling (H^+^, Na^+^, K^+^, and Ca^2+^) draws current from the electron transport pumps and thus consumes O_2_. Figure 4B shows the estimated ATP/O_2_ calculated using the following assumptions: i) Total O_2_ cost only includes O_2_ used for oxidative phosphorylation and O_2_ used to power cation cycles. The O_2_ consumed by the electrode is assumed to be negligible at these flux rates. Assuming the average excess O_2_ utilization (waste) shown in Figure 4C is representative for each calcium load, this relationship was used to correct for total O_2_ utilization shown in Figure 4A to remove O_2_ used to run futile cation cycles during oxidative phosphorylation. This relationship was approximated from the actual O_2_ use and the theoretical amount for the given bolus of ADP. Figure 4D shows how excess O_2_ utilization correlates strongly with duration of oxidative phosphorylation, which supports the idea that the O_2_ waste during oxidative phosphorylation occurs at a relatively constant, calcium load specific, and predictable rate. Lastly, Figure 4E shows that after a threshold, calcium load begins to impair oxidative phosphorylation rates and forces mitochondria to phosphorylate ADP at a slower rate relative to when calcium is low or absent. 

In addition, CsA tends to lower this duration back towards baseline, yet it did not impact the apparent or estimated ATP/O_2_ ratio. This effect of CsA is intriguing and is not likely to be related to its effect on the permeability transition phenomenon (i.e., pore gating). The effect is still present at low calcium loads, albeit very subtly. An alternative explanation of this effect ties into the role CsA plays in modulating mitochondrial ultrastructure [5]. Cryo–EM imaging reveals that CsA leads to a more condensed cristae network and presumably enhances energy transduction rates, and this observation lines up with the shorter durations of oxidative phosphorylation. All that said, the inhibitory role calcium plays during oxidative phosphorylation is becoming clearer and is still a potential target for ischemia/reperfusion injury and other metabolic–related disorders.

## 9. Concluding Remarks

How exactly do calcium phosphate precipitates impair oxidative metabolism? Do they directly or indirectly affect mitochondrial ultrastructure? Are the observed ultrastructural changes causal to the reduced capacity of calcium loaded mitochondria to produce and export ATP? These precipitates have been seen in rather tame cell culture conditions [88], but do they exist in cardiac mitochondria in living cardiac tissue? They have been found in infarcted cardiac tissue [89], but EM processing artifacts cannot be ruled out. As experimental techniques are limited, computational modeling is necessary to answer these questions related to metabolic functional capacity and ultrastructural features. The results presented herein reveal that the inhibition of oxidative phosphorylation is a calcium related phenomenon and is not caused by a direct decoupling of energy metabolism. In the calcium overloaded state, oxidative phosphorylation becomes rate limited by a yet to be determined mechanism that point towards either enzyme activity changes [1], ultrastructural modifications [2,5], or a combination of the two. As these questions are quite challenging to answer using today’s technology, detailed biophysical modeling of this phenomenon is the next best approach. Fortunately, several promising models [2,90,91,92] are available to establish a solid foundation from which to pursue the answer to these questions.

## Data Availability

The data presented in this study are available from the corresponding author upon request.
